# Pan-cancer analysis of systematic batch effects on somatic sequence variations

**DOI:** 10.1186/s12859-017-1627-7

**Published:** 2017-04-11

**Authors:** Ji-Hye Choi, Seong-Eui Hong, Hyun Goo Woo

**Affiliations:** 1 Department of Physiology, Ajou University School of Medicine, 164 Worldcup-ro, Yeongtong-gu, Suwon, South Korea; 2 Department of Biomedical Science, Graduate School, Ajou University, Suwon, South Korea

**Keywords:** Batch effect, TCGA, Pan-cancer, Mutation

## Abstract

**Background:**

The Cancer Genome Atlas (TCGA) is a comprehensive database that includes multi-layered cancer genome profiles. Large-scale collection of data inevitably generates batch effects introduced by differences in processing at various stages from sample collection to data generation. However, batch effects on the sequence variation and its characteristics have not been studied extensively.

**Results:**

We systematically evaluated batch effects on somatic sequence variations in pan-cancer TCGA data, revealing 999 somatic variants that were batch-biased with statistical significance (*P* < 0.00001, Fisher’s exact test, false discovery rate ≤ 0.0027). Most of the batch-biased variants were associated with specific sample plates. The batch-biased variants, which had a unique mutational spectrum with frequent indel-type mutations, preferentially occurred at sites prone to sequencing errors, *e.g.*, in long homopolymer runs. Non-indel type batch-biased variants were frequent at splicing sites with the unique consensus motif sequence ‘TTDTTTAGTT’. Furthermore, some batch-biased variants occur in known cancer genes, potentially causing misinterpretation of mutation profiles.

**Conclusions:**

Our strategy for identifying batch-biased variants and characterising sequence patterns might be useful in eliminating false variants and facilitating correct interpretation of sequence profiles.

**Electronic supplementary material:**

The online version of this article (doi:10.1186/s12859-017-1627-7) contains supplementary material, which is available to authorized users.

## Background

The Cancer Genome Atlas (TCGA) is a comprehensive database that includes multi-layered genome profiles collected from more than 30 cancer types, including genomic mutations, mRNA and miRNA, DNA copy number, DNA methylation and protein expression profiles. Large-scale collection of data inevitably generates batch effects. For example, processing steps ranging from sample collection to data generation are performed on different days using different lots of reagents or at different sites, potentially introducing batch effects into the data profiles. To compile the TCGA data, human tumour samples were collected from hundreds of Tissue Source Sites (TSS) and sent to two different Biospecimen Core Resources (BCRs), which processed quality-checked and stored specimens along with patients’ clinical information. On the other hand, genomic data were generated from multiple Genome Sequencing Centers (GSCs) and the Genome Characterization Centers (GCCs). These diverse data processing steps were barcoded with a unique sample name, which included the IDs of the TSS, participant, sample, vial, portion, analyte, plate and GSC (http://cancergenome.nih.gov/).

Previously, batch effects in genomic data have been addressed in diverse platforms including microarrays, RNA-seq and Exome-seq data [1–10]. In TCGA, systematic biases in variable batches have also been reported [11]. MBatch, a web-based analysis tool (http://bioinformatics.mdanderson.org/tcgabatcheffects), has been developed to investigate batch effects comprehensively. However, most of these studies are applicable only to continuous variables such as gene expression levels, but not to mutation data. Moreover, the overall patterns or characteristics of the sequence variant calls affected by batch effect have not been investigated extensively. Thus, in this study, we systematically evaluated batch effects on sequence variants identified from whole-exome data of TCGA. By examining barcodes that can introduce batch effects (*e.g.*, TSS, Plate ID, GSC, etc.), we identified 999 batch-biased variants from pan-cancer data. Systematic errors were generated by platform-dependent sequencing reactions, as well as batch effects due to different sampling conditions. By comparing mutational spectra of the batch-biased variants with the unbiased variants, we observed that batch-biased variants preferentially occurred at the sites prone to sequencing errors. Moreover, we found that the batch-biased variants had unique sequence patterns that could be recognised and eliminated from the data. Our systematic evaluation of batch-biased variants might be helpful in diminishing false-positive calls from the large-scale and heterogeneous TCGA profiles.

## Methods

### Data collection and processing

Of the 34 cancer types in TCGA (legacy level2, date 2016-05-24), we collected data from 20 cancer types which have more than 200 samples per cancer type. These included 7,502 tumor samples from 65 mutation annotation format (MAF) datasets, which identified somatic mutations in cancers using various analysis pipelines and/or sequencing platforms. Of the selected MAF files, those with extremely high frequency of indels (>500,000), no indels, or sample sizes less than 100 were filtered out. Finally, we used 46 MAF datasets from 19 cancer types for our analysis (Additional file 1: Figure S1 and S2; Additional file 2: Table S1).

Of the barcodes encoded in the sample names, we tested those with sufficient batch size (greater than 5) for each barcode ID, including the IDs for TSS, plate, and GSC. The BCR ID and date of shipment were also included in the barcodes. The batches (TSS, plate, or GSC) were obtained from the sample barcode IDs, and ‘date of shipment’ and ‘BCR ID’ were obtained from the clinical information and TCGA batch code table (https://github.com/saketkc/tcga-python/tree/master/TCGA/data), respectively. In addition, we also evaluated batch effects by clinical features such as patient’s age, gender, race, smoking history, ethnicity, tumor size, tumor grade, tumor stage, histological type, and country.

Additional annotation of mutations, including functional category, genomic region, and coding strand, were performed using the ANNOVAR software [12]. The flanking sequences of mutation sites were obtained using R library ‘Biostrings’. Homopolymer runs of each nucleotide were calculated by counting the number of continuous identical nucleotides (A, T, G, and C) within the flanking sequences of a variant.

### Estimation of batch-biased variant calls

To estimate batch effects on sequence variations, the number of samples with a variation in the tested barcode ID and other IDs *vs.* the number of samples without a variation were compared by Fisher’s exact test for each variation and each barcode ID. Filtering out the barcode IDs with mutation frequency < 3 in each MAF file, batch-biased variants were identified with a stringent statistical significance threshold of P-value < 0.00001. Then, if the two barcode IDs had more than 75% of the same batch-biased variants, the barcode ID with the highest frequency of batch-biased variants was chosen as representative.

### Phylogenic tree analysis

The pairwise distance from sequences of all mutation sites was evaluated using R library ‘phangorn’. The phylogenic tree was constructed using Neighbour-Joining tree estimation method.

### Consensus sequence and motif analysis

The position-specific consensus sequence of variants was evaluated using sequence-logo viewer Weblogo 3 with default parameters [13]. *De novo* motif sequence for batch-biased variants at splicing sites was identified using Homer with default parameters [14].

## Results

### Identification of batch-biased sequence variants in TCGA data

**Fig. 1:**
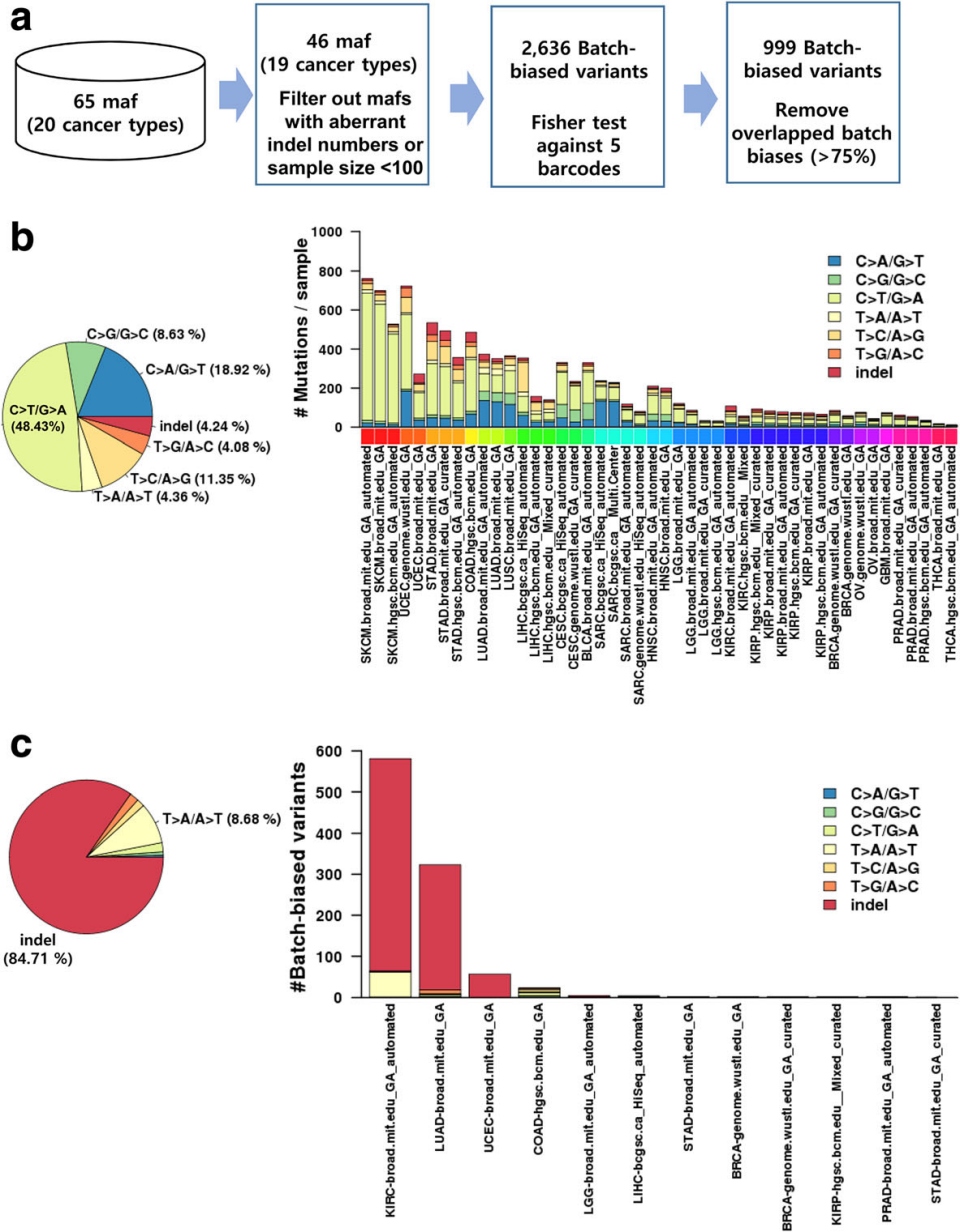
Identification of batch-biased variants. **a**. A workflow to identify batch-biased variants. **b**. Distribution of mutation types in TCGA data (*left*). Frequency of mutations in each MAF dataset according to mutation types (*right*). **c**. Distribution of mutation types of batch-biased variants (*left*). Frequency of batch-biased mutations in each MAF dataset according to mutation type (*right*)

In this study, we used 46 MAF datasets from 19 cancer types obtained from TCGA; the datasets were filtered as described in *Methods* (Fig. 1a). A total of 1,695,949 somatic sequence variants were included in the overall dataset. The mutation frequencies for each MAF dataset were highly variable, ranging from 10.33 to 761.52 mutations per sample. Skin cutaneous melanoma (SKCM) had the highest mutation rate (23.04%), whereas thyroid carcinoma (THCA) had the lowest mutation rate (0.36%) (Fig. 1b). Overall, C > T/G > A transition was the most frequent mutation type (49.46%), whereas the T > G/A > C transversion was the least frequent (3.82%) (Fig. 1b).

To determine possible batch effects on the sequence variant calls, we monitored the association of the mutation frequencies with batches prepared using different processing and data generation steps. Batch effects were estimated by applying Fisher’s exact test to each batch ID, as described in *Methods*. This analysis revealed 2,636 somatic sequence variants (false discovery rate; FDR ≤ 0.0027) associated with non-biological batches. Ultimately, 999 variants with representative batch IDs were determined as potential batch-biased variants (see Fig. 1a).

Most of the batch-biased variants were indel-type mutations (88.28%) (Fig. 1c, *left*). The frequencies of batch-biased variants varied considerably among the cancer types. The kidney renal clear cell carcinoma (KIRC, broad.mit.edu__IlluminaGA_automated_DNA_sequencing_level2.maf) had the most frequent batch-biased variants (*n =* 581), and LUAD had the second most frequent variants (*n =* 323). Batch-biased variants were found only in the 10 cancer types: KIRC, LUAD, UCEC, COAD, LGG, LIHC, STAD, BRCA, KIRP and PRAD (Table 1 and Fig. 1c, *right*). All the batch effects were related to the barcodes ‘plate’ (18 out of 19) or ‘TSS’ (1 out of 19). In particular, plate ID 0886 in KIRC (*n =* 577) and plate ID 0928 in LUAD (*n =* 313) had the most frequent batch-biased variants (Additional file 2: Table S2). To determine whether biological batches could be detected by our method, we also examined several clinical features, including the patient’s age, gender, race, smoking history, ethnicity, tumour size, tumour grade, tumour stage, histological type and country. However, none of the clinical features exhibited batch effects (data not shown), suggesting that our analysis can selectively identify non-biological batch effects.

**Table 1:** The list of biased batch IDs

Cancer type	#Total Variants	MAF	Batch ID	#Biased Variants
KIRC	25,372	KIRC-broad.mit.edu_GA_automated	plate_id ~ 0886	577
		KIRC-broad.mit.edu_GA_automated	plate_id ~ 0966	4
LUAD	188,695	LUAD-broad.mit.edu_GA	plate_id ~ 0928	313
		LUAD-broad.mit.edu_GA	plate_id ~ 1040	5
		LUAD-broad.mit.edu_GA	plate_id ~ 0969	4
		LUAD-broad.mit.edu_GA	plate_id ~ 2284	1
UCEC	52,789	UCEC-broad.mit.edu_GA	plate_id ~ A10C	56
		UCEC-broad.mit.edu_GA	plate_id ~ A10M	1
COAD	106,434	COAD-hgsc.bcm.edu_GA	plate_id ~ 1719	22
		COAD-hgsc.bcm.edu_GA	tss_id_AA	1
LGG	45,851	LGG-broad.mit.edu_GA_automated	plate_id ~ A289	4
		LGG-broad.mit.edu_GA_automated	plate_id ~ 1705	1
LIHC	71,536	LIHC-bcgsc.ca_HiSeq_automated	plate_id ~ A12Z	3
STAD	135,407	STAD-broad.mit.edu_GA	plate_id ~ 2340	2
	142,498	STAD-broad.mit.edu_GA_curated	plate_id ~ 2340	1
BRCA	84,908	BRCA-genome.wustl.edu_GA_curated	plate_id ~ A071	1
	45,607	BRCA-genome.wustl.edu_GA	plate_id ~ A188	1
KIRP	15,061	KIRP-hgsc.bcm.edu__Mixed_curated	plate_id ~ 1252	1
PRAD	23,023	PRAD-broad.mit.edu_GA_automated	plate_id ~ 2114	1

**Fig. 2:**
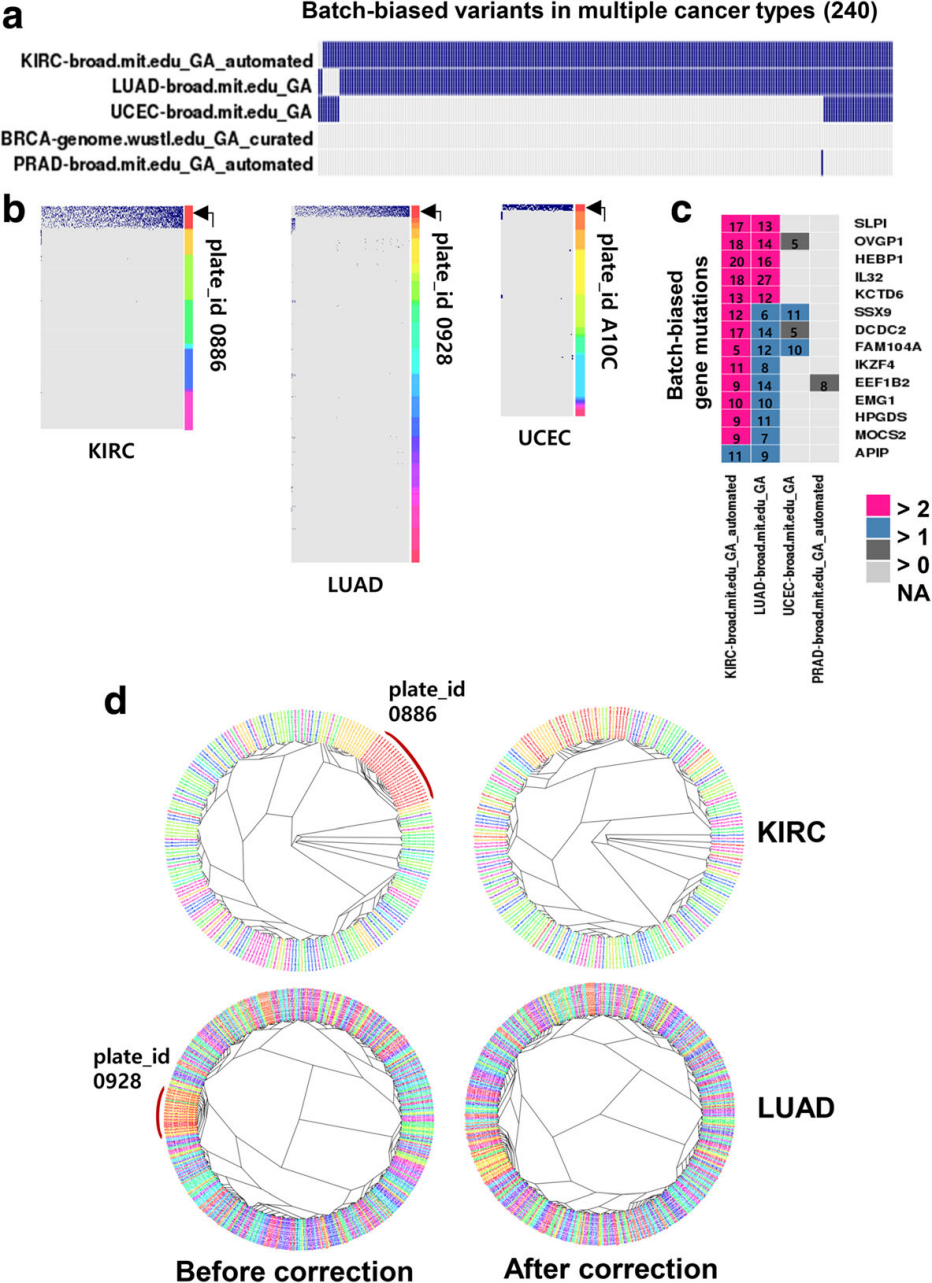
Recurrent batch-biased variants across cancer types. **a**. Heatmap shows 240 recurrent batch-biased variants in the MAF file. **b**. Batch-dependent occurrence of batch-biased variants is shown for KIRC, LUAD and UCEC datasets, respectively. Barcodes of plate IDs in each sample are indicated by different colours. **c**. Gene-level frequency of recurrent batch-biased variants (*n =* 14). The heatmap colours indicate the significance of batch-biased calls, *i.e.,* -log_10_(*P-value*), where P was calculated using Fisher’s exact test. **d**. Phylogenic tree analyses for KIRC (*upper*) and LUAD (*lower*) are shown. The phylogenic trees between the mutation profiles before correction (*left*) and after correction (*right*) are compared. The corrected mutation profiles are generated by removing the indicated batch-biased variants in each data. Barcodes of plate IDs in each sample are indicated by different colours

Of the 999 batch-biased variants, 240 (24%) variants occurred recurrently across different cancer types, indicating that batch biases were not related to cancer types (Fig. 2a). These recurrent batch biases were identified particularly in KIRC, LUAD, and UCEC with the plate IDs 0886 (*n =* 237), 0928 (*n =* 233) and A10C (*n =* 38), respectively (Fig. 2b). We also analysed the mutation frequencies at the gene level, revealing 14 genes with recurrent batch-biased mutations: *SLPI*, *OVGP1*, *HEBP1*, *IL32*, *KCTD6*, *SSX9*, *DCDC2*, *FAM104A*, *IKZF4*, *EEF1B2*, *EMG1*, *HPGDS*, *MOCS2,* and *APIP* (Fig. 2c). Based on these observations, we propose that the mutation frequencies of these genes might be overestimated by the batch-biased error calls, especially in KIRC, LUAD, and UCEC data.

In addition, to evaluate possible effects of mutation similarity of the samples on the batch effects, we performed phylogenic tree analysis on KIRC and LUAD data that had the most frequent batch-biased variants. This analysis revealed that the samples harboring batch-biased variants were clustered together, indicating that these samples had similar mutation profiles (Fig. 2d). However, the mutation profiles excluding the batch-biased variants did not cluster together. Thus, we could rule out the possibility that those batch-associated variants are the result of the similar mutation profiles among the samples.

### Comparison of mutation spectrum of the batch-biased and unbiased variants

**Fig. 3:**
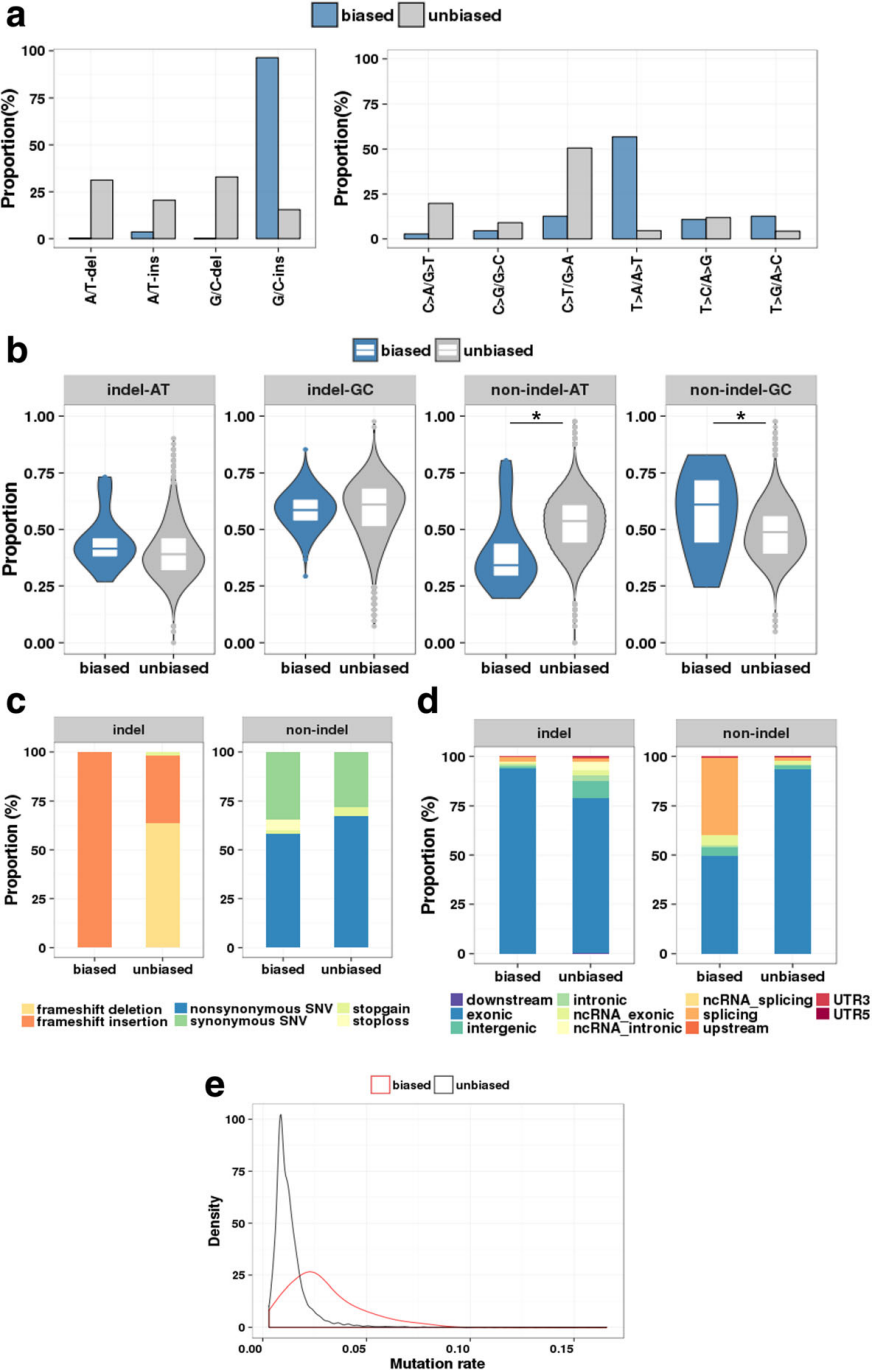
Comparison of distributions of batch-biased and unbiased variants. **a**. Distribution of indel types (*left*) and non-indel types (*right*) for the batch-biased variants (blue) and unbiased variants (*grey*). **b**. Distribution of GC content in each mutation type (*i.e.*, indel–AT, indel–GC, non-indel–AT and non-indel–GC) for batch-biased variants (blue) and unbiased variants (*grey*). Ratio of GC content was calculated using the 20 nucleotide sequences flanking the variant sites. (* *P <* 0.001.) **c**. Distribution of mutation types according to protein function for the batch-biased and unbiased calls with indel-type (*right*) and non-indel–type (*left*) mutations. **d**. Distribution of genome coordinates in relation to gene position for batch-biased variants and unbiased variants. **e**. Distribution of mutation rate in each MAF dataset for batch-biased variants (*red*) and unbiased variants (*black*)

Next, to delineate the overall characteristics of the batch-biased variants, we compared the mutation spectrum of the batch-biased variants with that of other unbiased variants (*n =* 1,695,223). The batch-biased variants had frequent G/C insertions (96.26%), whereas the unbiased variants did not (Fig. 3a). Of the substitution mutations, the batch-biased variants had frequent T > A/A > T mutations (56.75%), whereas the unbiased variants had frequent C > T/G > A mutations (50.57%), revealing a large difference in mutation spectrum between the biased and the unbiased variants (Fig. 3a).

We also evaluated the possible association of GC content with the batch-biased variant calls. By calculating the percentage of GC content in the 20 nucleotides flanking the mutation sites for each of the mutation types (AT/GC and indel/non-indels, *i.e.*, indel–AT, indel–GC, non-indel–AT and non-indel–GC), we found that the batch-biased non-indel–GC variants had significantly higher GC contents than the unbiased variants (*P* = 9.66 × 10^−3^, Student’s *T*-test). Conversely, the batch-biased non-indel–AT variants showed significantly lower GC content than the unbiased variants (*P* = 3.82 × 10^−10^, Student’s *T*-test). By contrast, the frequency of indel-type batch-biased variants was not related to the neighbouring GC content levels (Fig. 3b). In addition, batch-biased indels had frequent frameshift insertions, whereas the unbiased indels had frequent frameshift deletions (Fig. 3c). Of the non-indel variants, the batch-biased variants had more frequent stop-loss mutations than the unbiased non-indel variants (*P* = 1.38 × 10^−5^, Fig. 3c). These findings imply that generation of batch-biased variants can largely be attributed to neighbouring sequences with preferential mutation types and characteristic GC contents.

Previous genomic landscape studies of the sequence mutations exhibited that the mutation rates vary considerably among genomic locations [15]. When we evaluated the distribution of the genomic coordinates of the variants in relation to gene positions, we found that the batch-biased variants occurred frequently at splicing sites, although the unbiased variants did not (*P* = 1.62 × 10^−48^, Fig. 3d). This result implies the preferential occurrence of erroneous batch-biased variant calls at splicing sites. Furthermore, we found that the batch-biased variants had higher mutation rates than the unbiased variants (Fig. 3e). Taken together, our results suggest that batch-biased erroneous sequence variants have unique genomic features and preferentially occur at the positions prone to sequencing errors. Thus, sequencing errors might be more frequent when the sample batches were prepared from lower-quality tissues, either due to inadequate processing of the tissue or errors in data generation.

### Homopolymer runs are associated with batch-biased variants

**Fig. 4:**
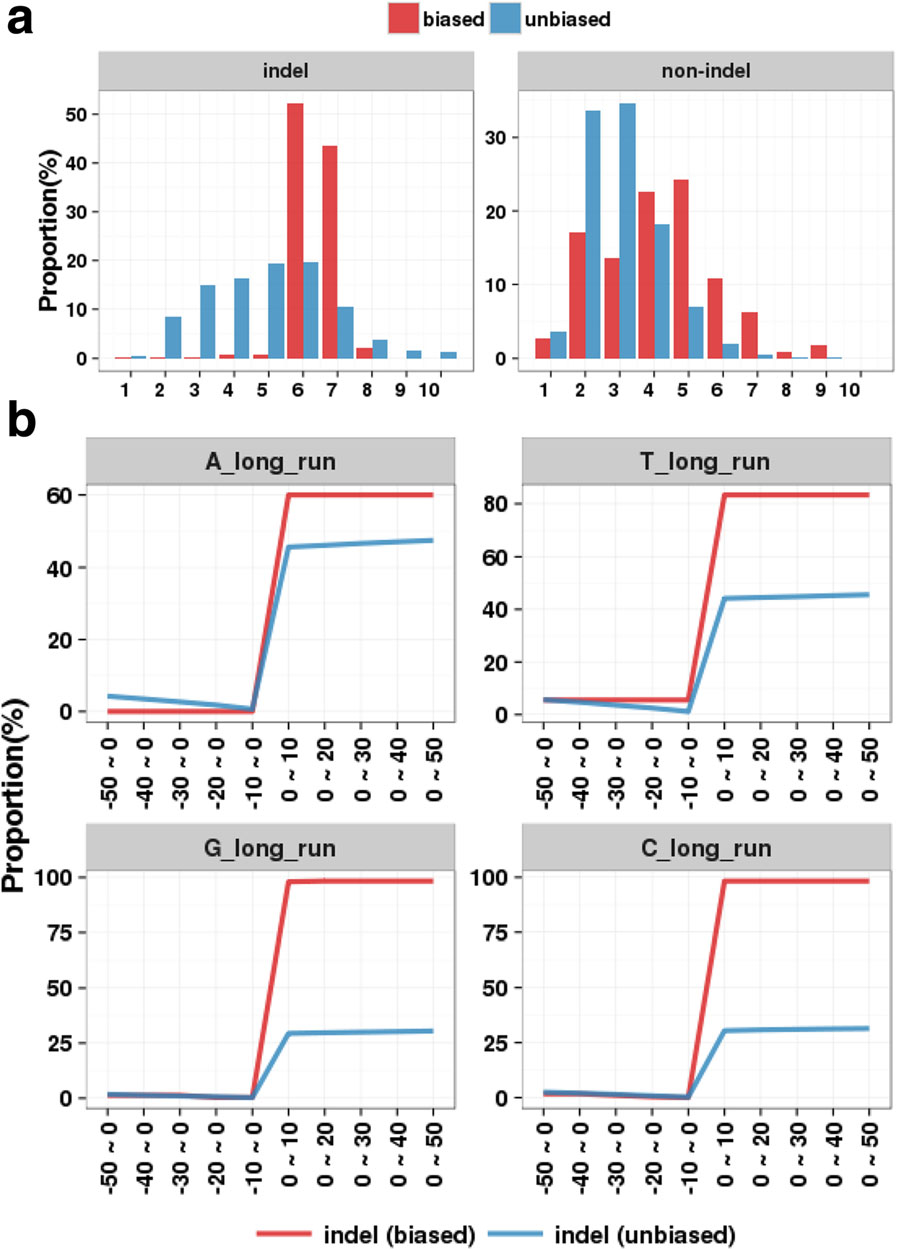
Homopolymer runs in flanking sequence of code-biased mutations. **a** Distribution of homopolymer run within the 50 nucleotide sequences flanking the batch-biased variants and unbiased variants with indel-type (*left*) and non-indel–type (*right*) mutations. **b** Distribution of the long (over 5) homopolymer runs within flanking regions of different window sizes for batch-biased and unbiased variants

Sequencing errors, particularly indels, occur frequently at repeated homopolymer sequences [16–19]. With this in mind, we investigated whether the batch-biased variants are associated with the repeated sequences. For each nucleotide type (A, T, G and C), we calculated the number of homopolymer runs which was determined based on the maximum length of repeated nucleotides in the 50 nucleotides in the left and right regions from the variant sites. Remarkably, we observed that the distribution of the number of homopolymer runs in batch-biased variants was right-shifted relative to that in the unbiased variants (Indel; *P* < 2.2 × 10^−16^, non-indel; *P* = 2.78 × 10^−11^, Fig. 4a), indicating that the batch-biased variants were more frequent in longer homopolymer runs. When we examined the distribution of homopolymer runs for each mutation type, batch-biased variants frequently had longer homopolymer runs with more than five repeated nucleotides (Fig. 4a and Additional file 1: Figure S3). We also examined the distribution of homopolymer runs with different window sizes of flanking sequence regions. Most of the homopolymer runs were found in downstream regions within 10 base pairs of the variant sites (Fig. 4b). This indicates that the batch effect on the variant call is strongly associated with the homopolymer run. Because our observations revealed the sequence patterns affected by the flanking regions, we next examined the position-specific consensus sequence distribution using a sequence-logo viewer Weblogo 3 [13]. This analysis revealed the consensus sequence of homopolymer runs at the flanking regions (Additional file 1: Figure S4) which were previously identified as the sites likely prone to sequencing error [17, 19].

### Batch-biased variants occur frequently at splicing site

**Fig. 5:**
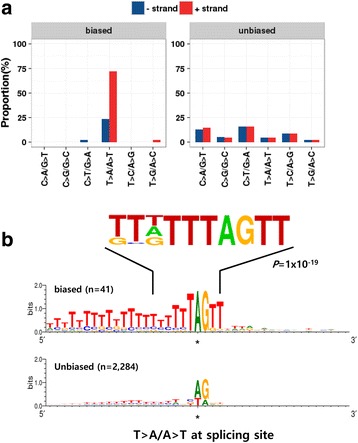
Batch-biased non-indel variants occur preferentially at splicing site. **a**. Distribution of mutations types in batch-biased (*left*) and unbiased (*right*) non-indel variants at splicing sites. Mutation frequencies in each plus and minus strand are indicated by red and blue bars, respectively. **b**. The sequence-logo plots show the consensus sequence in the 20 nucleotide regions flanking the batch-biased (*upper*) and unbiased (*lower*) T > A/A > T variants at splicing sites. Consensus motif sequence was identified using Homer, a *de novo* motif-finding tool

In Fig. 3d, we showed that batch-biased non-indels were frequently observed at splicing sites. Moreover, we observed that the batch-biased variants had frequent T > A/A > T mutations, particularly in the sense strand, implying selection bias for batch-biased calls at splicing sites (Fig. 5a). Therefore, we sought to identify position-specific consensus sequences at splicing sites using a sequence-logo viewer. As shown in Fig. 5, the batch-biased but not the unbiased T > A/A > T variants had a clear consensus sequence of the dinucleotide ‘AG’ with upstream long T polymers and the dinucleotide ‘TT’ in the following downstream sequence (Fig. 5b). This finding suggests that batch-biased variants at splicing sites occur preferentially at splicing acceptor sites (‘AG’) with upstream polypyrimidine tracts in the intronic region. In addition, *de novo* motif analysis using Homer [14] revealed the consensus motif sequence ‘TTDTTTAGTT’ for the batch-biased T/A variants at splicing sites (*P* = 1 × 10^−19^). This information might facilitate development of methods for eliminating possible batch-biased variants at splicing sites.

### Batch-biased variants in significantly mutated genes (SMGs)

**Fig. 6:**
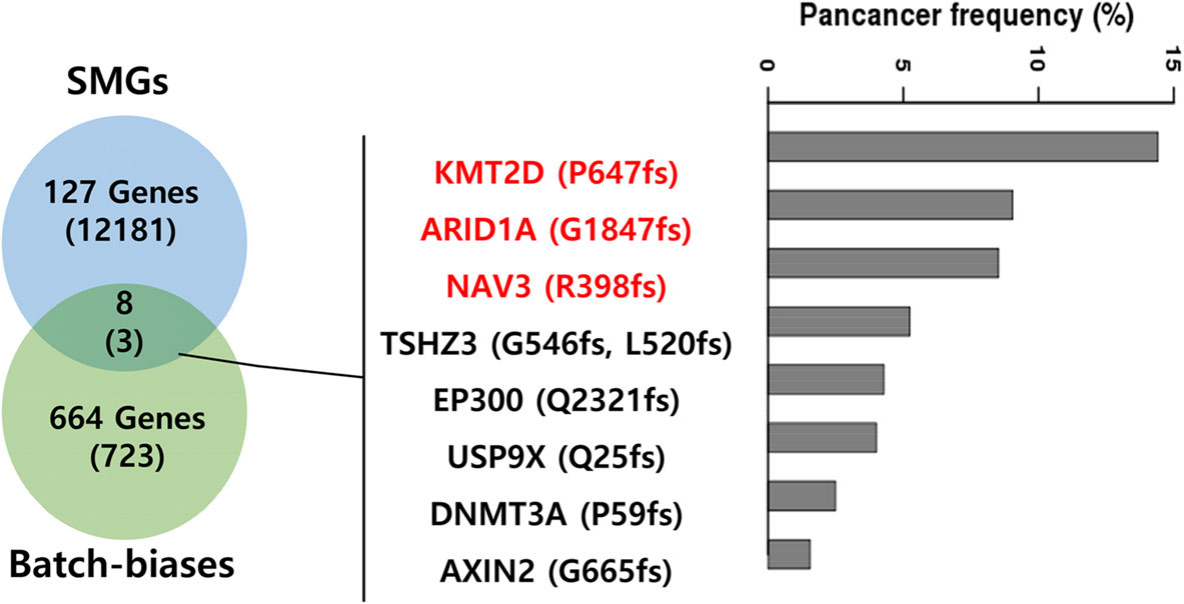
Batch-biased variants in the significantly mutated genes (SMGs). The Venn diagram shows the overlap between SMG genes and genes containing batch-biased variants. The number of variants in each gene is indicated in round brackets (*left*). Genes with position-matched batch-biased variants are indicated in red. Frequency of gene mutations in the pan-cancer data is shown in a bar plot (*right*)

The presence of batch-biased variants in cancer-driver genes could induce serious errors in data interpretation. As a proof-of-concept, we examined the significantly mutated genes (SMGs, *n =* 127), previously identified in an analysis of 12 cancer types as genes that might play driver functions in cancer progression [20]. Of the SMGs, eight (*KMT2D*, *ARID1A*, *NAV3*, *TSHZ3*, *EP300*, *USP9X*, *DNMT3A,* and *AXIN2*) overlapped with the set of genes harbouring batch-biased variants (Fig. 6 and Additional file 1: Table S3). In particular, *KMT2D*, *ARID1A* and *NAV3* had mutation sites that exactly matched the batch-biased variants. Moreover, these genes had relatively high mutation rates in the pan-cancer data (*KMT2D*, 14.41%; *ARID1A*, 9.04%; *NAV3*, 8.52%), which might be overestimated due to the erroneous batch-biased variant calls, although this remains to be validated (Fig. 6). Together, these findings suggest that batch effects on the sequence variants should be considered carefully.

## Discussion

In this study, by performing a pan-cancer analysis of exome sequencing data from TCGA, we evaluated possible batch effects on somatic mutation calls and identified 999 potential batch-biased variants. Batch-biased sequence variants were frequently found in specific cancer types: KIRC, LUAD and UCEC. Most of the batch effects were identified from plate IDs 0886 and 0928, implying possible problems with tissue quality on these plates.

Mutation spectrum analysis of batch-biased calls revealed sequencing platform–dependent, error-prone features of the batch-biased variants. Our results imply that the sample batches with poor tissue quality may lead to batch-biased sequencing errors which might preferentially occur at the sites prone to sequencing error rather than randomly.

In fact, sequencing platform–dependent substitution errors, particularly in the Illumina sequencing systems, have been reported previously [16, 21–23]. Frequent sequencing errors are associated with higher GC content and the presence of long G/C homopolymers [24]. To reduce false calls, many of variant calling methods have been developed that filter variants based on minimum depth of coverage, masking of repetitive sequence regions and trimming of poor-quality bases from the ends of reads [25–27]. However, although batch-biased sequencing errors occurred preferentially at sequencing error–prone sites, the batch-biased errors differed significantly from the platform-dependent sequencing errors. We demonstrated that indels were more frequent than substitution mutations in the batch-biased variants, whereas Illumina sequencers generate more substitution-type errors more often than indel type [16]. Comparison of batch-biased variants with the unbiased variants also revealed distinct sequence patterns and mutation spectra between them. These results imply that the biased variants occur preferentially at sites prone to platform-dependent sequencing error, but they also have unique genomic features independent of previously established error-prone sites.

Interestingly, we found that the batch-biased variants were frequent at splicing sites (see Fig. 3), where they may be associated with homopolymer runs at flanking regions. Indeed, analysis of the flanking sequences of the batch-biased splicing sites revealed consensus sequences: upstream long T homopolymer in the intronic region and downstream dinucleotide ‘TT’. This finding is consistent with previous studies reporting that the rates of erroneous sequencing increase at long homopolymer runs [16–19]. Nearly all introns of the U2 type are spliced by the major spliceosome and flanked by GT-AG splice site dinucleotides [28]. The polypyrimidine tracts of GT-AG and GC-AG splice sites have C-rich and T-rich sequences, respectively [29]. Thus, batch-biased T > A/A > T variants might occur preferentially at U2-type GC-AG splicing sites. Indeed, preferential sequencing errors at splicing sites have been reported previously in multiple studies. Sequencing errors are prone to occur at the G base of 3′ splice dinucleotides because of the suppression of G after incorporation of A in the dye-terminator sequencing reaction [30]. Moreover, the ‘G after A’ problem is further enhanced by the polypyrimidine tract that precedes the acceptor AG. Sequencing errors at homopolymer runs of T and C can be generated by polymerase slippage. However, because mutations at splicing sites are implicated in a number of cellular processes and diseases [31], preferential batch-biased mutations at splicing sites may lead to misinterpretation of the functional effects of mutation profiles.

In addition, as a proof-of-concept, we demonstrated that some SMG genes harbour these batch-biased variants with relatively higher mutation rates (see Fig. 6). This result implies that batch-biased variants might occur even at cancer genes, emphasising those batch-biased variants should be considered carefully in mutation data analysis.

TCGA data are still processing update, correcting erroneous variant calls. However, many previous studies using the legacy TCGA data might be affected by the batch biases. Therefore, our findings regarding batch bias effect might be helpful in validating or re-analyzing studies using these legacy data.

## Conclusion

Our strategy for identifying batch-biased variants and characterising sequence patterns might be useful for eliminating false variant calls, and could thus help to interpret sequence profiles correctly.

## Additional files

Additional file 1: Figure S1. Frequency of indels and non-indels in each MAF dataset. Distribution of mutation frequency across MAFs for indel variants (*upper*) and non-indel variants (*lower*). *, MAF datasets with no indels are indicated. **Figure S2.** Number of MAF data for each cancer type. Bar plot shows the number of MAF datasets available for each TCGA cancer type. **Figure S3.** Homopolymer runs in the flanking sequences of the batch-biased indels. Distribution of long (red) and short (blue) homopolymer runs for each altered nucleotide (A, T, G and C) within the 50 nucleotide sequences flanking each variant, shown for batch-biased indel variants (*upper*) and unbiased indel variants (*lower*). **Figure S4.** Consensus sequences in flanking sequences of batch-biased indels. The sequence-logo plots show consensus sequences in the 15 nucleotide flanking regions of the batch-biased (upper) and unbiased (lower) variants for each of the altered nucleotides (A, T, G and C). (DOCX 602 kb)

Additional file 2: Table S1. List of the MAFs used in the analysis. **Table S2.** List of 999 batch-biased variants in TCGA data. **Table S3.** List of batch-biased genes among the SMGs. (XLSX 111 kb)

## Abbreviations

BRC: Biospecimen Core Resource
BRCA: Breast invasive carcinoma
COAD: Colon adenocarcinoma
GCC: Genome Characterization Center
GSC: Genome Sequencing Center
KIRC: Kidney renal clear cell carcinoma
KIRP: Kidney renal papillary cell carcinoma
LGG: Lower grade glioma
LIHC: Liver hepatocellular carcinoma
LUAD: Lung adenocarcinoma
MAF: Mutation annotation format
PRAD: Prostate adenocarcinoma
SKCM: Skin cutaneous melanoma
SMG: Significantly mutated gene
SNP: Single-nucleotide polymorphism
STAD: Stomach adenocarcinoma
STAD: Stomach adenocarcinoma
TCGA: The Cancer Genome Atlas
THCA: Thyroid carcinoma
TSS: Tissue Source Sites
UCEC: Uterine corpus endometrial carcinoma
