# Supplementary material for: Pan-cancer analysis of systematic batch effects on somatic sequence variations
Source: BMC Bioinformatics. 2017 Apr 11;18:211. doi: 10.1186/s12859-017-1627-7 (PMC5387285; doi:10.1186/s12859-017-1627-7)
Supplement: Supplementary file 1 — Frequency of indels and non-indels in each MAF dataset. Distribution of mutation frequency across MAFs for indel variants (upper) and non-indel variants (lower). *, MAF datasets with no indels are indicated. Figure S2. Number of MAF data for each cancer type. Bar plot shows the number of MAF datasets available for each TCGA cancer type. Figure S3. Homopolymer runs in the flanking sequences of the batch-biased indels. Distribution of long (red) and short (blue) homopolymer runs for each altered nucleotide (A, T, G and C) within the 50 nucleotide sequences flanking each variant, shown for batch-biased indel variants (upper) and unbiased indel variants (lower). Figure S4. Consensus sequences in flanking sequences of batch-biased indels. The sequence-logo plots show consensus sequences in the 15 nucleotide flanking regions of the batch-biased (upper) and unbiased (lower) variants for each of the altered nucleotides (A, T, G and C). (DOCX 602 kb) [file 12859_2017_1627_MOESM1_ESM.docx]

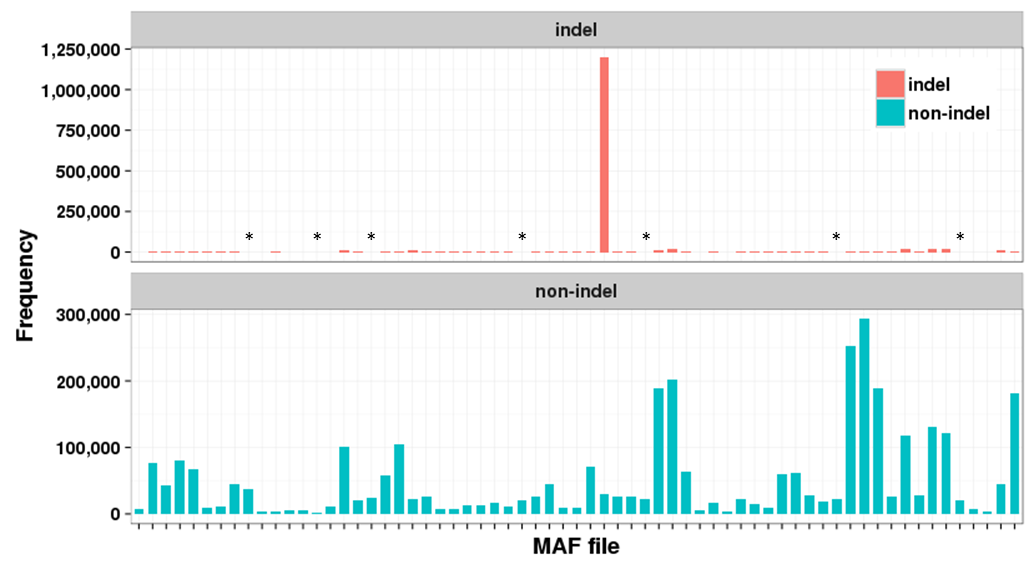
**Supplementary Figures**

**Figure S1. Frequency of indel and non-indel mutations in each MAF file**


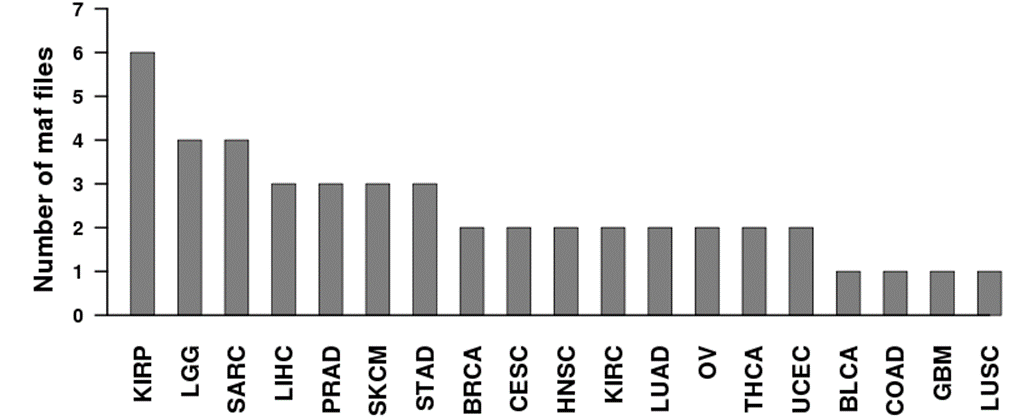


**Figure S2. Number of MAF files for each cancer type**


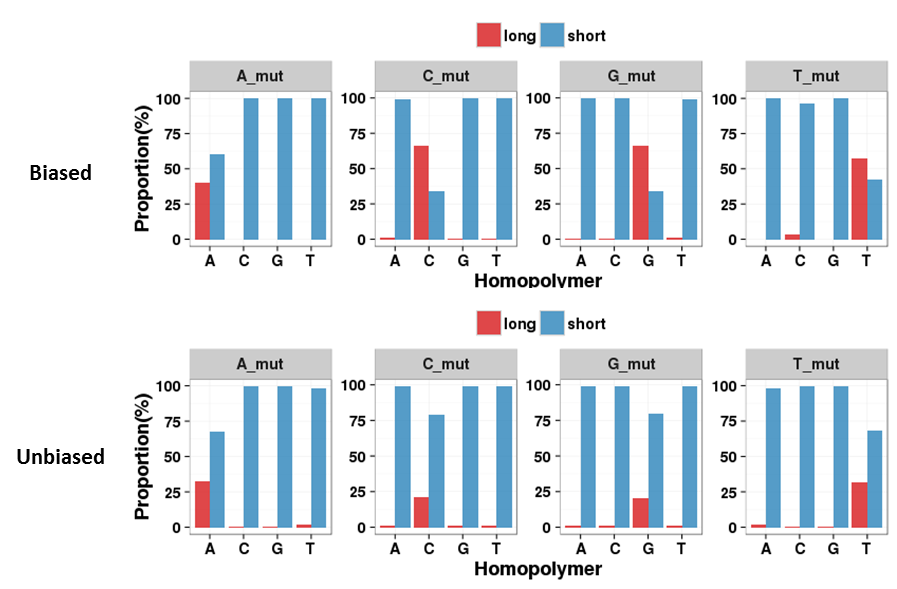


**Figure S3. Homopolymer runs in flanking sequences of batch-biased indels**

**Figure S4. Consensus sequences at flanking sequences of batch-biased indels**
